# Flexible ureteroscopy versus laparoscopy for the treatment of patients who initially presented with obstructive pyelonephritis

**DOI:** 10.12669/pjms.323.9938

**Published:** 2016

**Authors:** Selcuk Sahin, Berkan Resorlu, Mithat Eksi, Bekir Aras, Arda Atar, Volkan Tugcu

**Affiliations:** 1 Selcuk Sahin, Bakirkoy Dr. Sadi Konuk Training and Research Hospital, Department of Urology, Istanbul, Turkey; 2 Berkan Resorlu, Department of Urology, Ondokuz Mayis Univercity, Faculty of Medicine, Samsun, Turkey; 3 Mithat Eksi, Bakirkoy Dr. Sadi Konuk Training and Research Hospital, Department of Urology, Istanbul, Turkey; 4 Bekir Aras, Department of Urology, Dumlupinar Univercity, Faculty of Medicine, Kutahya, Turkey; 5 Arda Atar, Bakirkoy Dr. Sadi Konuk Training and Research Hospital, Department of Urology, Istanbul, Turkey; 6 Volkan Tugcu, Bakirkoy Dr. Sadi Konuk Training and Research Hospital, Department of Urology, Istanbul, Turkey

**Keywords:** Flexible ureteroscopy, Laparoscopic ureterolithotomy, Proximal ureteral stone, Obstructive pyelonephritis, Transperitoneal

## Abstract

**Objective:**

To compare the safety and effectiveness of flexible ureteroscopy (F-URS) with transperitoneal laparoscopic ureterolithotomy (TPLU) in cases of obstructive pyelonephritis secondary to large proximal ureteral stones.

**Methods:**

A series of 42 patients presenting with obstructive pyelonephritis due to proximal ureteral stones larger than 1.5 cm were included from April 2006 to February 2015 in this comparative study. After drainage of pyonephrosis and resolution of sepsis, 22 patients treated with TPLU (Group I), and 20 patients were treated with F-URS (Group II). Preoperative patient and stone characteristics, procedure-related parameters and clinical outcomes were assessed for each group.

**Results:**

It was seen that both methods were effective in the treatment of large proximal ureteral stones. However TPLU provided a higher stone- free rate (100% vs 80%. p=0.043) and lower retreatment rate. There was no difference between the groups for the operative time and complication rate. On the other hand, patients treated with F-URS had less postoperative pain (p=0.008), a shorter hospital stay (p<0.001) and a faster return to daily activities (p<0.001).

**Conclusions:**

The results of our study show that both F-URS and TPLU are safe and effective surgical procedures for treatment of large proximal ureteral stones after controlling obstructive pyelonephritis. However, TPLU has a higher stone-free rate with comparable operating time and complication rate as compared to F-URS. On the other hand F-URS has the advantages of less postoperative pain, shorter hospital stay and faster return to daily activities.

## INTRODUCTION

Urosepsis due to ureteral obstruction by stone impaction is a life-threatining condition which usually require urgent drainage of the collecting system.^1^,^2^ Emergent collecting system decompression with percutaneous nephrostomy tube or double-J stent and proper antibiotic therapy are the essential steps of the treatment before stone surgery.^3^-^7^ After the initial treatment, available techniques include shock wave lithotripsy (SWL), ureteroscopy (URS) using flexible or semirigid devices, laparoscopic ureterolithotomy and open surgery.

The most popular techniques are SWL and URS for the treatment of upper ureteral calculi less than 1 cm in size, because of low morbidity and acceptable efficacy of these methods. But SWL has a lower success rate compared with URS when it is used for the ureteral stones larger than 1cm.^8^ Various studies in the literature have investigated the success of SWL, URS and laparoscopic ureterolithotomy in the treatment of large proximal ureteral stones.^7^-^9^ But the best treatment option is still controversial, and to the best of our knowledge those techniques have not been compared in cases of obstructive pyelonephritis.

Therefore, we compared the safety and efficacy of TPLU and F-URS in patients who initially presented with pyelonephritis secondary to large ureteral stone.

## METHODS

The study received institutional ethics committee approval in accordance with the Declaration of Helsinki. We performed a retrospective analysis of 42 patients presenting with obstructive pyelonephritis due to proximal ureteral stones larger than 1.5 cm from April 2006 to February 2015. All patients were initially given parenteral antibiotics and underwent nephrostomy tube placement. After drainage of pyonephrosis and resolution of sepsis, 22 patients were treated with TPLU (Group I), and 20 patients were treated with F-URS (Group II) for stone removal.

Stone size was measured by using the longest axis of stone viewed on preoperative imaging. Patients who had multiple stones, or younger than 18 years of age were excluded. The data were collected prospectively and analyzed retrospectively. The treatment method was chosen by considering the preference of the patients, after the advantages and disadvantages of the techniques were discussed with the patients.

The diagnosis of obstructive pyelonephritis was based on the symptoms of sepsis, the presence of flank pain, radiological findings (confirmation of ureteral stone and concomitant hydronephrosis), and laboratory data, such as bacteriuria and leukocyturia. The drainage of obstructive pyelonephritis was carried out mainly by antegrade percutaneous nephrostomy. Nephrostomy tube using a 8-Fr pigtail stent was carried out under ultrasound and fluoroscopic guidance. All patients received proper antibiotic treatment according to urine culture preoperatively.

All operations were performed by single experienced surgeon (VT) under general anesthesia. An indwelling JJ ureteral stent was placed and then the nephrostomy catheter was removed at the end of the procedure in all patients. On postoperative day one plain film was taken to check the position of the JJ stent and clinically insignificant residual fragments (< 4 mm). Ureteral stents were removed 4 to 6 weeks after surgery. Intravenous Urography was performed at three months of follow-up. Subsequent follow-up of the patients was sone at 6 months and then annually.

Patient demographics, stone characteristics, and procedure related parameters including success rate, operation time, VAS (visual analog scale), hospital stay, and complications were noted and compared for each group. Modified Clavien Grading System was used for classification of the complications.

Statistical analyses were performed using Number Cruncher Statistical System (NCCS) 2007 Statistical Software (Kaysville, Utah, USA) program. Data were analyzed using descriptive statistical methods (mean, standard deviation, median, frequency and rate) as well as the normal distribution showing comparisons between groups of variables Independent Samples Test; variables did not show normal distribution, Mann-Whitney-U test was used. In the comparison of qualitative datas Yates Continuity Correction and Fisher’s exact test was used. P < 0.05 was considered statistically significant.

## RESULTS

### Patient and Stone Characteristics

The present study included 26 (61.9%) men and 16 (38.1%) women. Group I consisted of 22, and group II consisted of 20 patients. There was no significant difference between the groups of patients for age, gender, stone size, stone density and stone site; however, there was a significant difference for body mass index (BMI). The mean BMI were 25.9±2.3 kg/m^2^ and 27.8±1.3 kg/m^2^ in group I and II, respectively (p=0.002). The patient demographics and stone characteristics of the groups are shown in Table-I.

**Table-I:** Patient and stone characteristics.

	*TPLU Group (Group I)*	*F-URS Group (Group II)*	*p value*
No. patients (%)	22	20	
Male / Female	14 / 8	12 / 8	
Mean age±sd, years	47.3±10.0	49.0±8.7	0.556
Mean stone size±sd, mm	19.9±3.2	19.6±2.6	0.698
Mean BMI, kg/m^2^	25.9±2.3	27.8±1.3	0.002*
Stone side (right/left)	12 / 10	10 / 10	1
Mean stone density (HU)	1000.0±293.9	1184.65± 144.2	0.013*

* Significant at 0.05 level.

### Operative Findings

The mean operation time was 74.1±12.2 minutes and 78.1±6.4 minutes in group I and II, respectively (p=0.192). The stone-free rate was 100% in Group I, and 80% in Group II after a single procedure (p=0.043). The retreatment rate was significantly higher in group II than group I (20% vs 0%, respectively). Any of the patients in either group did not have bleeding requiring blood transfusion and the mean blood loss was 58.8 mL in Group I. Intraoperative findings of the patients are summarized in Table-II.

**Table-II:** Comparison of intraoperative and postoperative data.

	*TPLU Group (Group I)*	*F-URS Group (Group II)*	*p value*
Mean operation time, min	74.1±12.2	78±6.4	0.192
Stone-free rate	100%	80%	0.043*
Re-treatment rate	0%	20%	
Mean hospital stay, days	4.2±1.2	2.1±1.1	0.001*
Return to normal activity, days	13.3±1.7	9.0±1.6	0.001*
*VAS score*			
Day 0	6.4±1.3	5.1±1.6	<0.008*
Day 1	4.3±1.0	3.6±1.2	0.093
Complications	5 (22.7%)	7 (35%)	0.591
*Grade I*			
Mucosal injury	-	1	
Stent related dyscomfort	2	2	
Ileus	3	1	
*Grade II*			
Ureteral perforation	-	1	
Fever	-	2	

* Significant at 0.05 level

### Postoperative Findings and Complications

The mean hospital stay was 4.2±1.2 days in the TPLU group and 2.1±1.1 days in F-URS group (p<0.001). The mean VAS score obtained 6 hours after surgery was 6.4±1.3 in TPLU versus 5.1±1.6 in F-URS (p=0.008) and on the first postoperative day mean VAS was 4.3±1.0 in TPLU versus 3.6±1.2 for F-URS2 (p=0.093). Time to return daily routine activities was 13.3±1.7 days in Group I, and 9.0±1.6 days in Group II (p<0.001).

The complications were classified according to modified Clavien classification system, and are presented in Table-II. No major complications such as septic shock or death were reported in either treatment group. The overall complication rate was 22.7% in Group I, and 35% in Group II. This difference of complications between two groups was not statistically significant (p=0.591).

## DISCUSSION

F-URS and TPLU are the most common used treatment modalities for the management of proximal ureteral stones larger than 1.5 cm. In this study, we showed that TPLU had a higher stone-free and lower retreatment rate compared with F-URS. On the other hand, patients treated with F-URS had less postoperative pain, a shorter hospital stay and a faster return to daily activities. But surprisingly, two techniques did not differ significantly in terms of complication rates and operation times.

Obstructive pyelonephritis secondary to ureteral stones requires emergency drainage of the renal collecting system with percutaneous nephrostomy tube or double-J stent.^1^,^2^ An early diagnosis, urgent drainage of the collecting system, and proper treatment with antibiotics are essential steps for the management of these patients. An obstructed and infected kidney can progress to life-threatining conditions such as urosepsis and septic shock despite urgent drainage and appropriate antibiotic therapy, a mortality rate of 2% may still be expected.^10^ After drainage of pyonephrosis and resolution of sepsis, definitive treatment for the ureteral stone should be planned. However, the best treatment option for those cases is still controversial, especially for large and impacted stones.

Available techniques include SWL, semirigid or flexible URS, laparoscopic ureterolithotomy and open surgery. Selecting the best technique for the treatment of stones should favor not only the minimally invasive and comfortable method but also the most effective procedure.^11^ Although SWL has been accepted as the first treatment option for proximal ureteral stones smaller than 1 cm with low complication and high stone-free rates, a general reluctance exists to choose this non-invasive technique for large upper ureteral stones.^12^ In a recent study, Lopes Neto et al. prospectively compared the effectiveness of URS, SWL and laparoscopic ureterolithotomy for the treatment of large upper ureteral stones and found a low success rate of 37.5%, with SWL.^13^

F-URS is another option with high success rate but has some difficulties in the management of impacted large upper ureteral stones. Difficulties about ureteroscopic methods was associated with accessing stones secondary to ureteral lesions such as edema or polyps and tortuous ureter.^11^ Kumar et al. reported a success rate of 86% in patients with impacted ureteral stones.^14^ In another study, Lee et al. reported a retreatment rate of 42% with F-URS in cases having large proximal ureteral stones.^15^ In a recent study, including 103 patients with stone size more than 1.5 cm, Ko et al. compared the URS and laparoscopic ureterolithotomy.^16^ The stone clearence rate was significantly higher in the laparoscopy group (100%) as compared to URS (77%) group.

Laparoscopic ureterolithotomy can be performed through a retroperitoneal or transperitoneal route. In this study, transperitoneal approach was used in all cases. The most important advantages of transperitoneal route over the retroperitoneal one is that it provides the good working space and identification of anatomical landmarks are easier. Our study showed that the stone-free rate was significantly greater in the TPLU group (100%) as compared to the F-URS (80%) group, which indicated TPLU was a perfect option for proximal ureteral stones. However, the patients treated with F-URS had less postoperative pain, a shorter hospital stay and a faster return to daily activities.

The complication rate after F-URS (22%) was lower than after TPLU (35%), with no statistically significant difference. There was no important infectious complication such as sepsis or septic shock and deaths in each group. Two patients in group II had fever on the first day after operation, which resolved with antibiotic treatment. Our results suggest that post-drainage F-URS and TPLU are safe and feasible techniques.

### Limitation of the study

Our study is a retrospective analysis of a single institution with a limited number of cases. Therefore, our findings must be confirmed by large prospective randomized trials to define the optimal management of these patients.

## CONCLUSION

The results of our study show that both F-URS and TPLU are safe and effective surgical procedures for treatment of large proximal ureteral stones after controlling obstructive pyelonephritis. However, TPLU has a higher stone-free rate with comparable operating time and complication rate as compared to F-URS. On the other hand F-URS has the advantages of less postoperative pain, shorter hospital stay and faster return to daily activities.
